# A one-step route to solubilised, purified or functionalised single-walled carbon nanotubes†
†Electronic supplementary information (ESI) available: Supplementary figures. See DOI: 10.1039/c5ta03561a



**DOI:** 10.1039/c5ta03561a

**Published:** 2015-07-23

**Authors:** A. J. Clancy, J. Melbourne, M. S. P. Shaffer

**Affiliations:** a London Centre for Nanotechnology , Department of Chemistry , Imperial College London , South Kensington , SW7 2AZ , UK . Email: m.shaffer@imperial.ac.uk

## Abstract

Reductive dissolution is a promising processing route for single walled carbon nanotubes (SWCNTs) that avoids the damage caused by ultrasonication and aggressive oxidation whilst simultaneously allowing access to a wealth of SWCNT functionalisation reactions. Here, reductive dissolution has been simplified to a single one-pot reaction through the use of sodium naphthalide in dimethylacetamide allowing direct synthesis of SWCNT Na^+^ solutions. Gram quantities of SWCNTs can be dissolved at concentrations over 2 mg mL^–1^. These reduced SWCNT solutions can easily be functionalised through the addition of alkyl halides; reducing steric bulk of the grafting moiety and increasing polarisability of the leaving group increases the extent of functionalisation. An optimised absolute sodium concentration of 25 mM is shown to be more important than carbon to metal ratio in determining the maximum degree of functionalisation. This novel dissolution system can be modified for use as a non-destructive purification route for raw SWCNT powder by adjusting the degree of charging to dissolve carbonaceous impurities, catalyst particles and defective material, before processing the remaining SWCNTs.

## Abstract 2

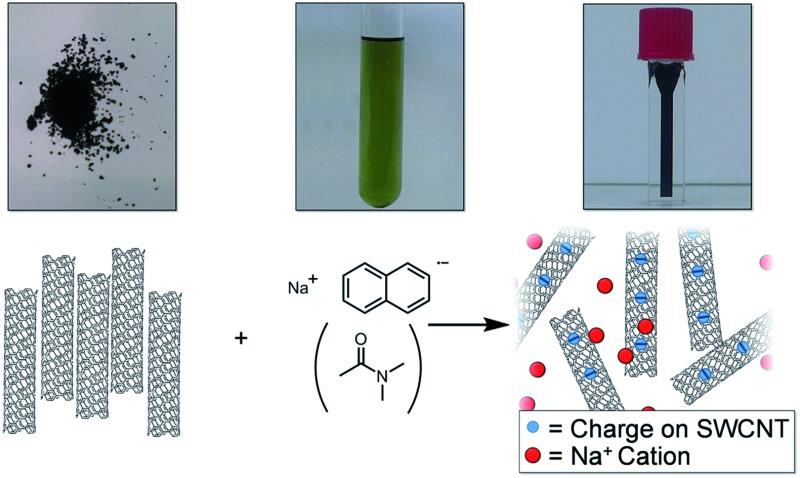
The use of *N*,*N*-dimethylformamide as a solvent for carbon nanotube reduction and dissolution allows simple and effective dissolution and purification.

## 1. Introduction

Since their discovery, single walled carbon nanotubes (SWCNTs) have been of great interest due to their high strength,^1^ stiffness, surface area, and aspect ratio, along with their interesting optoelectronic^2^ and thermal properties.^3^ However, due to their high surface energy^4^ (0.5 eV nm^–1^), as-produced SWCNTs form aligned bundles^5^ which are mechanically^6^ and electronically^7^ inferior to individualised nanotubes in most applications. Functionalisation of bundled SWCNTs generally occurs at the exposed exterior surface limiting both the total level and the uniformity of functionalisation. As-synthesised carbon nanotubes also routinely contain impurities both in the form of non-nanotube carbons (amorphous and graphitic carbon, and short defective nanotubes) and residual catalyst particles, often contained within graphitic shells and CNT caps.^8–10^ For these reasons, the superlative properties of SWCNTs can generally only be exploited after purification, individualisation, and stabilisation of the nanotubes in a solvent.

The most common route to dispersing SWCNT bundles uses intense sonication and stabilisation of the resulting suspensions with aqueous surfactant solutions,^11^ amphiphilic or conjugated polymers,^12^ DNA,^13^ or amidic/amidinic solvents such as *N*-methyl pyrrolidone (NMP).^14–16^ Sonication is difficult to scale and both damages the SWCNT sp^2^ framework and reduces aspect ratio;^17^ although alternative, scalable high shear mechanical force dispersion techniques have recently been applied to carbon nanotubes, damage is still a significant issue.^18^ In any case, the solution formed through sonication still consists primarily of smaller bundles of nanotubes; although the individualised fraction can be separated by ultracentrifugation, it is a low yield, costly, and time consuming process. Oxidation by refluxing nanotubes in nitric acid (or other oxidising conditions) can purify the sample,^19^ etching away carbonaceous impurities and dissolving metal contaminants, and introducing potentially useful carboxyl groups;^20^ however, the resultant nanotubes are heavily damaged in the process with carbons atoms removed from the framework,^21^ leading to large reductions in mechanical properties.^22^

An alternative route to SWCNT dissolution involves charging of the nanotubes leading to electrostatic repulsion of the resultant nanotubide anions^23^ (or nanotubium cations) allowing a spontaneous route to truly individualised solutions of nanotubes without damage to the sp^2^ carbon framework.^24^ Addition of SWCNTs to chlorosulfonic acid^25^ leads to spontaneous dissolution of the nanotubes^26^ through protonation of the SWCNT sidewalls (which can be deprotonated *via* addition to water)^27^ at very high concentration.^28^ However, chlorosulfonic acid is difficult to handle and is incompatible with many chemical species, limiting the options for organic functionalisation. Reductive charging can be accomplished using methodologies including direct electrochemistry,^29^ Birch reductions,^30^ addition of group 1 organometallics, and the use of charged organic electron transfer agents.^31^ In this latter case, dried SWCNTs are initially exposed to a charging agent, typically a group 1 metal with either liquid ammonia or a solution of an organic single electron transport agent such as naphthalene^32,33^ or 4,4′-di-*tert*-butylbiphenyl.^33^ The nanotubide can then be isolated by removal of the charging agent and solvent, and dissolved in one of a selection of polar aprotic solvents. It is worth noting that although widely used for this step, DMSO is thought to partially quench the nanotubide, methylating the nanotubes^34^ at high nanotubide charge densities. This approach to chemical reduction is typically a time consuming process, requiring several steps followed by high vacuum and/or elevated temperatures to remove the reduction solvent prior to dissolution in a suitable reaction solvent.

Beyond simple dissolution, the raised Fermi level of reduced SWCNTs can be used to access a host of nanotube functionalisation reactions,^35^ thought to proceed through a single electron transfer mechanism,^35,36^ similar to the reaction of sodium naphthalide with alkyl halides; the nanotubide reduces a grafting species R–X, which decomposes to an anionic leaving group X^–^ and the radical R˙ which can graft onto the SWCNT sidewall. Many compounds can be grafted allowing for a versatile array of functionalisation, including organohalides^37^ and disulfides^38^ for alkyl/arylation, bromine^39^ for halogenation, carbon dioxide^21^ for carboxylation, and epoxides^40^ and vinyl monomers^41^ for oligo/polymerisations from the SWCNT sidewall.

**Fig. 1:**
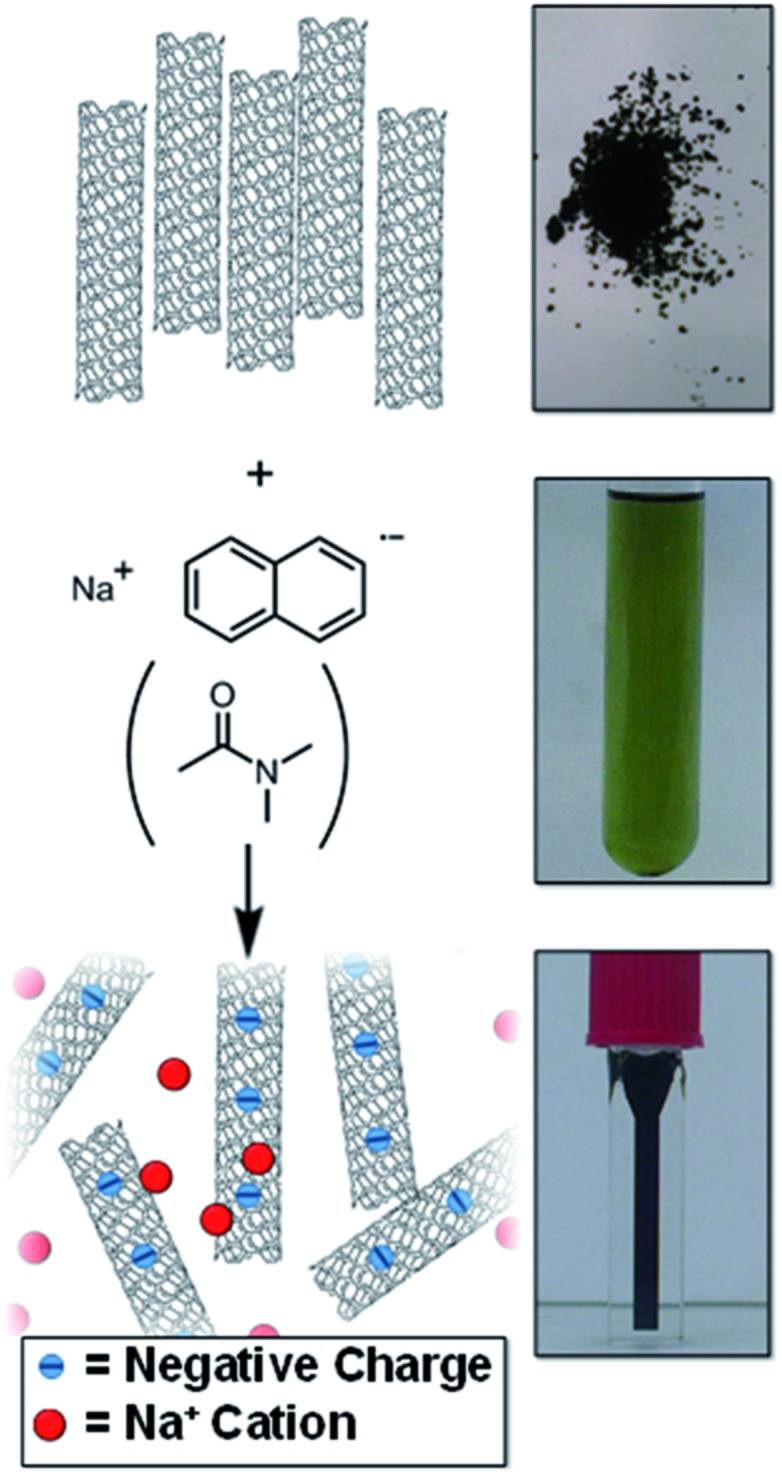
Schematic (left) and pictures (right) of raw SWCNT powder and NaNp solution in DMAc forming a solution of nanotubide.

There is a need to simplify and improve current SWCNT processing (dissolution, functionalisation, and purification) to accelerate work in the area. Herein, a simple SWCNT reduction system is proposed that can facilitate SWCNT dissolution (Fig. 1) and non-damaging purification; the factors which govern the extent of reductive grafting are examined to elucidate the mechanism and maximise the grafting density for future application.

## 2. Experimental

### 2.1. Materials

Elicarb single walled carbon nanotubes (PR929, batch 108511/g), supplied by Thomas Swan Ltd. were dried under vacuum (∼10^–2^ mbar) at 300 °C for 1 h and 16 h at room temperature before use. All other chemicals were purchased from Sigma Aldrich. Solvents were purchased as anhydrous grade and further dried with activated 4 Å molecular sieves for 2 days prior to use; additionally, THF and DMSO were degassed to completion *via* freeze–pump–thaw. Sodium (99.95%) was purchased as an ingot; naphthalene (99%) was dried under vacuum in the presence of P_2_O_5_. All work was done in a N_2_ glovebox using oven dried glassware.

### 2.2. Procedures

#### Sodium naphthalide stability tests

Sodium (5 mg) and naphthalene (28 mg) were added to 5 mL of solvent and stirred with a glass stirrer bar for 24 h. For pre-prepared sodium naphthalide tests, sodium (5 mg) and naphthalene (56 mg) were added to THF (10 mL) and stirred overnight with a glass stirrer bar. Excess naphthalene was used to ensure nonmetallic sodium was present. The THF was then distilled off, the crystals were allowed to cool to RT and solvent (5 mL) was added and stirred overnight with a glass stirrer bar.

#### Typical SWCNT reductive dissolution

A bulk solution of sodium naphthalide in DMAc was prepared by stirring sodium (50 mg) and naphthalene (278 mg) in DMAc (50 mL) using a glass stirrer bar. For high levels of charging at high SWCNT loadings, higher concentration solutions may be necessary. Sodium naphthalide solutions were used within a week of preparation. For a 1 mg mL^–1^ loading of SWCNTs charged to a C : Na ratio of 10 : 1, 19.2 mL of 1 mg_(Na)_ mL^–1^ sodium naphthalide was diluted with 80.8 mL of DMAc and added to dried SWCNTs (100 mg). The mixture was stirred with a glass stirrer bar overnight before pipetting into fluorinated ethylene propylene (FEP) centrifuge tubes (Oak Ridge) with PTFE tape sealing the cap thread and centrifuged at 10 000*g* for 30 min; solutions were then pipetted off by hand. Concentrations were measured by quenching 10 mL of solution by bubbling with dry oxygen for ∼20 min before filtering over a tared 100 nm pore PTFE membrane and washing with copious amounts of ethanol, DI water, and acetone, ensuring the sample did not dry out between washings. The sample was then dried at 150 °C for 3 hours and weighed. A membrane subjected to the same washing procedure using 10 mL of DMAc *in lieu* of the SWCNT solution returned the tared weight (within 0.1 mg).

#### Alkylation of reduced SWCNTs

A solution of reduced SWCNTs (10 mg SWCNT) was diluted with DMAc to the desired concentration. Alkyl halides were dried (activated 10× molecular sieves) and degassed (if liquid, *via* freeze–pump–thaw) and added to the SWCNT solution (1 molar eq. *vs.* Na) then stirred overnight with a glass stirrer bar, before bubbling with dry oxygen for ∼20 min followed by filtering over a 100 nm pore PTFE membrane and washing with copious ethanol, DI water, and acetone, ensuring the sample did not dry out between washings.

#### Typical SWCNT purification

Premade sodium naphthalide solution in DMAc was added to SWCNTs as above, but solutions were left to stand for 48 h without stirring. The mixture was pipetted into FEP centrifuge tubes with PTFE tape sealing the cap thread and centrifuging at 10 000*g* for 30 min; supernatant was decanted. Supernatant concentrations were measured as above. Residual purified nanotubes were exposed to a dry oxygen environment overnight and washed by stirring in ethanol, water and acetone, filtering over 100 nm PTFE membranes between steps. It should be noted that some impurities (*e.g.* residual catalyst particles) may be smaller than the membrane pore size thus the yields should be treated as a lower bound.

### 2.3. Measurements

UV-Vis spectra were taken with a Perkin Elmer Lambda 450 with an integration time of 0.5 s in an optical glass cuvette with a pathlength of 4 mm and screw-top lid with PTFE tape sealing the thread for air sensitive samples. When *A* > 1.0, the sample was diluted 10× and spectra values were multiplied by 10. SEM images and EDX spectra were taken using a Leo Gemini 1525 FEGSEM at an accelerating voltage of 10 keV for SEM, and 20 keV for EDX. TEM images were taken with a JEOL 2000 with an accelerating voltage of 100 keV. Raman spectra were taken with a ISA Jobin Yvon SPEX Raman exciting with a 25 mW 532 nm laser. Statistical Raman was performed with a Renishaw inVia micro-Raman Spectrometer with 633 nm laser. TGA measurements were taken with a Perkin Elmer Pyris 1 under N_2_ at 60 mL min^–1^ holding for 60 min at 100 °C before increasing the temperature at 10 °C min^–1^ to 800 °C. AFM was performed on samples drop-cast onto cleaned (H_2_SO_4_/H_2_O_2_) silicon wafers, dried, and soaked in ethanol and water. AFM micrographs were taken by tapping mode on a Digital Instruments Multimode VIII AFM with Nanoscope IV Digital Instruments AFM controller (Veeco) using Nanosensor tapping mode probes (Windsor Scientific).

## 3. Results and discussion

### 3.1. One-step reduction and dissolution

The key step in simplifying chemical reduction was to identify a stable reductant/solvent system using a nanotubide solubilising solvent, allowing the SWCNTs to both charge and dissolve in one scalable step. Known organic solvents for nanotubide include dimethyl sulfoxide (DMSO), *N*,*N*-dimethylformamide (DMF), and *N*-methyl-2-pyrrolidone (NMP). In addition, *N*-cyclohexyl-2-pyrrolidone (CHP), a related solvent to NMP previously identified as an optimum solvent for uncharged SWCNT dispersion *via* sonication,^15^ was tested. The reducing agent for the one-step system was selected from those proven to be successful in previous nanotubide literature. Direct (vapour) metal intercalation and Birch reductions were not considered compatible with a single-step process, whilst other organometallic reducing agents (such as BuLi) initiate undesirable side reactions, either with amide solvents, or by secondary alkylation of the nanotubes. As such, organic charge transfer agents were preferred and sodium naphthalide (NaNp) was selected as its reduction of SWCNTs is well studied,^23^ and it can be easily visually identified by a characteristic green colour. A strong reducing potential^42^ is desirable to maximise the charge density on the nanotubes, as long as solvent degradation is avoided.

**Fig. 2:**
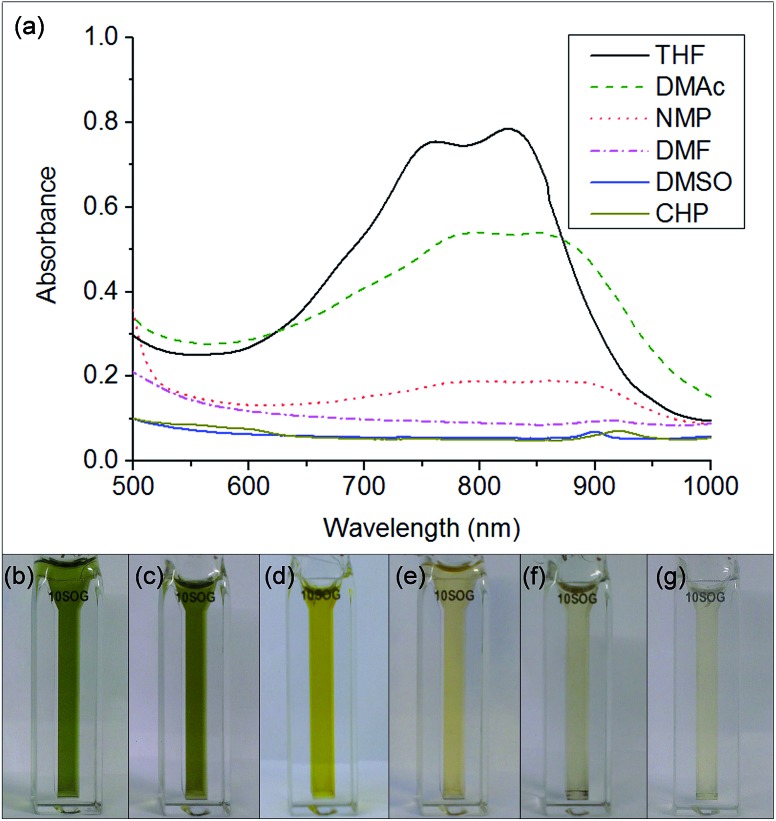
(a) UV-Vis spectra of equimolar sodium and naphthalene (of ∼1 mg_(Na)_ mL^–1^) added to a selection of solvents. (b–g) Cuvettes containing the Na + Np solutions of (b) THF (c) DMAc (d) DMF (e) NMP (f) DMSO (g) CHP.

**Scheme 1:**
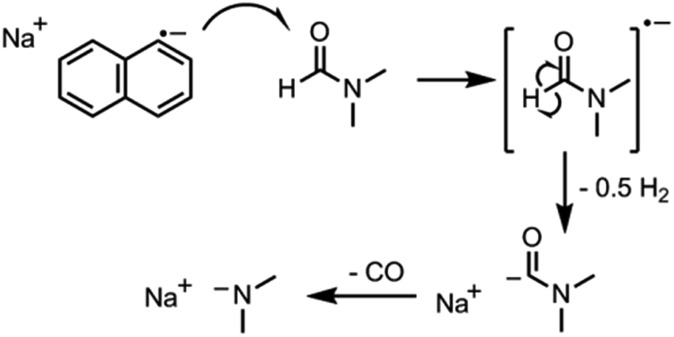
Proposed decomposition of DMF in the presence of sodium naphthalide in neat solvent.

When either equimolar sodium and naphthalene or pre-synthesised NaNp crystals were stirred into DMSO, DMF, CHP and NMP at 1 mg_(Na)_ mL^–1^, the solvents turned yellow/orange and increased in viscosity. The disappearance of napthalide's characteristic double peak (735 nm and 827 nm in THF, Fig. 2)^42^ in the UV-Vis spectra of these solutions was attributed to reaction with the solvents. The instability of NMP and CHP may relate to the opening of the strained ring. The degradation pathway of DMF in the presence of sodium metal is known^43^ and is initiated by reduction of DMF immediately followed by removal of the formic proton to form a metal formadyl species that can further degrade to the unstable and highly basic NaNMe_2_ which can decompose further DMF solvent molecules. A similar pathway was assumed to apply for the observed DMF degradation with NaNp (Scheme 1). It was proposed that replacement of the formic proton in DMF with a methyl group to form *N*,*N*′-dimethylacetamide (DMAc) would disrupt these pathways and prevent degradation. Addition of sodium and naphthalene to DMAc led to the formation of a green colour and full dissolution of sodium to produce 0.1 M solutions within 15 min stirring. The UV-Vis spectrum shows the expected naphthalide peaks at 757 and 850 nm; the small red-shift compared to the THF solution (22 nm) is attributed to the higher dielectric constant of DMAc, as seen for other charged organic complexes in varying solvents.^44^ The extinction coefficient in DMAc appears to be intrinsically lower than in THF (*ε*_761 nm(THF)_ = 30 200 m M^–1^, *ε*_798 nm(DMAc)_ = 26 200 m M^–1^, ESI 1†). The stability of the spectrum over time excludes any ongoing reaction between the naphthalide and the solvent; the solutions obey the Beer–Lambert law upon dilution excluding the presence of a naphthalide destructive impurity (*e.g*. trace water contamination). To illustrate the flexibility of this DMAc system, the stability of other known SWCNT single electron transport reducing agents, potassium naphthalide^31^ and sodium 4,4′-di-*tert*-butylbiphenylide,^33^ was also demonstrated (ESI 4†).

**Fig. 3:**
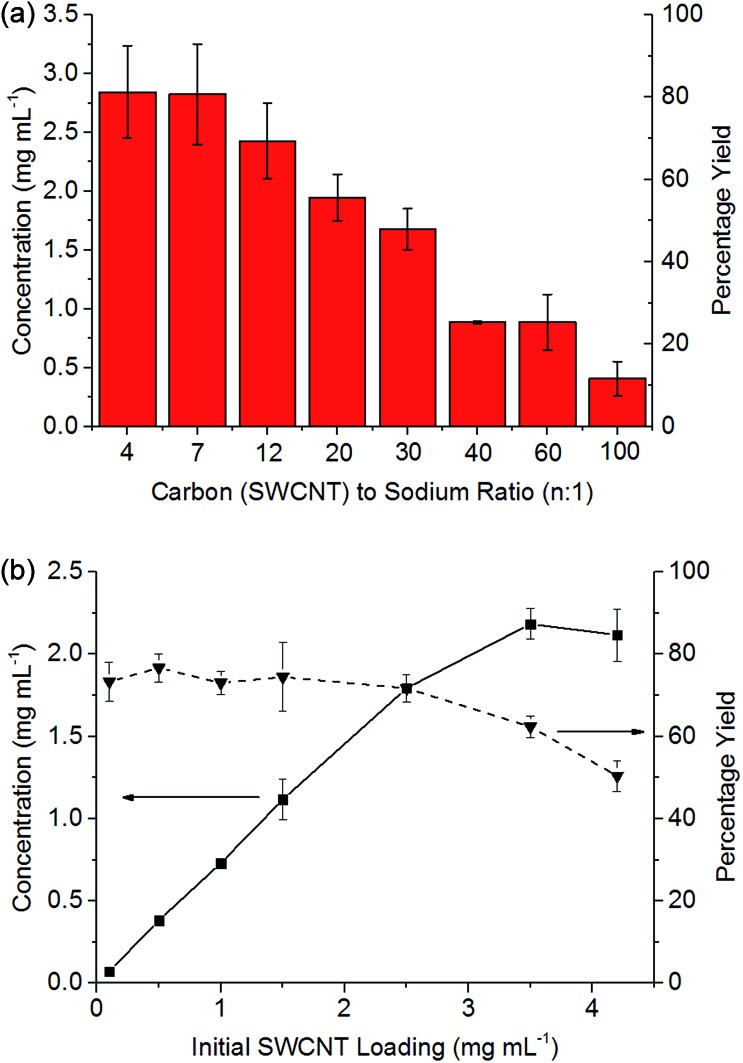
(a) Influence of C : Na ratio on the concentration of nanotubide solution starting with 3.5 mg mL^–1^ loading of SWCNT. (b) The yield and concentration of nanotubide solutions against initial SWCNT loadings with 20 : 1 C/Na. Error bars represent standard deviations of 3 repeats.

Stirring of dried SWCNT powder (Thomas Swan Elicarb) into a solution of sodium naphthalide in DMAc (NaNp/DMAc) led to rapid dissolution with a black solution of SWCNTs forming within minutes (Scheme 1), seen to be individualized SWCNTs by AFM (ESI 2†). At a set charging ratio, here C : Na = 20 : 1 (Fig. 3b), the concentration of nanotubide solution initially scales linearly with the loading of SWCNTs maintaining a yield between 73 and 77%, indicating that the incomplete dissolution is not due to saturation, but is due to an inherently insoluble fraction of ∼20% wt. At higher initial loadings of SWCNTs, the concentration does not increase further, causing the yield to drop as the solution appears to saturate, here at ∼2 mg mL^–1^.

At higher degrees of charge (*i.e.* lower C : Na ratios), the limiting solubility occurs at higher concentrations (Fig. 3a), although never at >80% yield for this SWCNT feedstock. At extremely high loadings (*e.g.* 6.5 mg mL^–1^, ESI 3†), the SWCNTs can be seen to form a gel. Increasing the degree of charge (*i.e.* lower C : Na ratios) with a fixed initial SWCNT loading (3.5 mg mL^–1^) leads to an increasing yield of SWCNTs in solution as expected due to increased coulombic repulsion; however, excessive charge is known to lead to ‘salting out’ effects^31^ in nanotubide solutions and polyelectrolytes in general. The concentrations here are greater than those demonstrated through NaNp charged nanotubide in DMSO (0.4 mg mL^–1^)^23^ and are on par with the sonication of predominantly bundles of SWCNTs.^45^ Recent research has shown speed mixing of nanotubide salts with a solvent and crown ether leads to higher concentrations (up to 52 mg mL^–1^), although these high concentration mixtures have not been shown to be fully individualised.^15^ The concentrations here (3.5 mg mL^–1^) are also lower than optimised sonication^46^ in aqueous surfactant solution (20 mg mL^–1^). It is worth reiterating, however, that concentrated sonicated dispersions consist primarily of bundles^47^ unlike spontaneously dispersed nanotubide solutions.^24^

### 3.2. Functionalisation of reduced SWCNT solutions

**Fig. 4:**
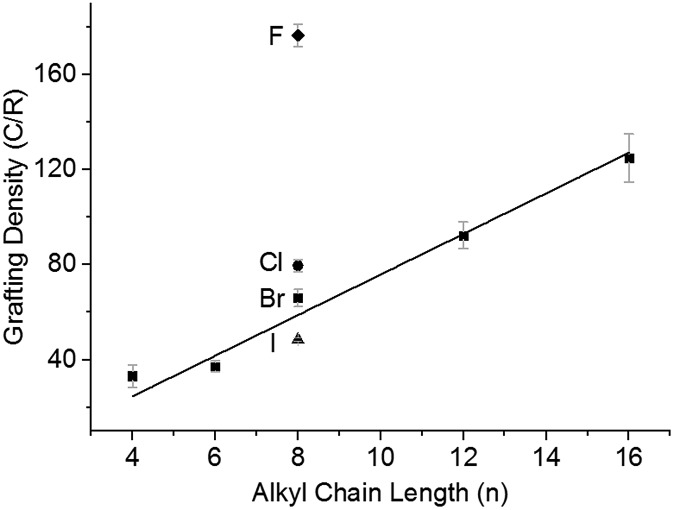
Grafting density of alkyl halides as a function of alkyl chain length (C_*n*_H_2*n*+1_Br) and halide; C_8_H_17_X where X = F (◆) Cl, (), Br (■) or I (▲) for 20 : 1 charged SWCNTs. Line fitted to alkyl bromide series.

Chemical modification of SWCNTs allows adjustment of solubility,^48^ wettability,^49^ and introduction of new functionalities. Nanotubide solutions synthesised by reduction and dissolution with NaNp/DMAc are capable of reacting with alkyl halides to graft organic moieties, providing a quick non-damaging and simple route to grafted SWCNTs from the raw powder. The functionalisation of nanotubide solutions in DMAc was studied on a model series of alkyl halides. Firstly, the effect of varying halide leaving group was explored by reacting NaNp/DMAc reduced SWCNTs, changed at 20 : 1 C : Na, with the 1-halide octanes (C_8_H_17_X; X = F, Cl, Br, I), followed by quenching with dry oxygen, filtering, and washing. Grafting density (alkyl groups per SWCNT carbon; lower numbers indicate higher levels of functionalisation) was calculated *via* TGA (ESI 8†). There is a distinct trend of increasing halide weight increasing the level of functionalisation (Fig. 4); similar to the effect seen for functionalisation of graphite intercalation compounds (GICs),^50^ which can be rationalised with the single electron transport mechanism.^36^ The increasing polarisability (and less negative reduction potentials)^51^ of alkyl halides along the series F < Cl < Br < I dictates the ease of radical anion formation through reduction by nanotubide, leading to a higher concentration of alkyl radicals formed for the heavier, polarisable alkyl halides with less negative reduction potentials, and a subsequently higher grafting density on the SWCNTs.

The steric bulk of the alkyl radical also affected the degree of functionalisation; as the reactivity of *n*-alkyl radicals do not vary significantly,^52^ any change in grafting density when varying alkyl length is attributed primarily to steric effects. On grafting a series of linear alkyl bromides (C_*n*_H_2*n*+1_Br where *n* = 4, 6, 8, 12, 16) to 20 : 1 charged NaNp/DMAc reduced SWCNTs, a linear decrease in grafting can be seen with increasing alkyl length (Fig. 4). This near linear trend of alkyl chain length *versus* grafting ratio has also been observed before for GICs.^50,53^

Even with a highly effective leaving group and short chain (C_4_H_9_Br), the grafting density remains below the 20 : 1 C/R that would correspond with 100% utilisation of the 20 : 1 charge used to reduce the system. Some charge will be lost creating radicals which react to form dimers,^53^ although previous work has demonstrated that the quantity of dimer produced is small.^35^ An alternative limit to the reaction occurs as the nanotubide loses charge during the reaction and the Fermi level drops eventually beneath the reduction potential of the alkyl halide. Related phenomena have been demonstrated previously *via* the reactivity of analogous reduced graphene sheets with metal salts.^54^

**Fig. 5:**
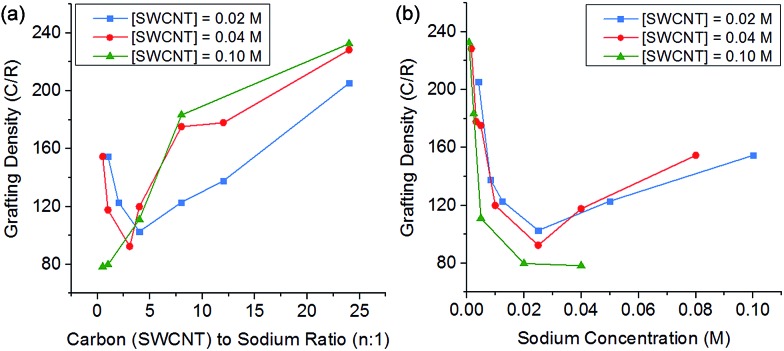
Grafting density of reduced SWCNTs added to 1-bromododecane *versus* (a) C : Na ratio, (b) sodium concentration, illustrating a shifting ideal stoichiometry but a stable sodium concentration.

Altering the degree of charge on the nanotubide by varying the C : Na ratio also impacts the degree of grafting. At low charge ratios, there is less charge available for functionalisation and grafting levels are low; however, at high levels of charge the degree of grafting also decreases (Fig. 5). This behaviour can be explained by polyelectrolyte ‘salting out’ discussed earlier, leading to bundling of the SWCNTs reducing accessible surface area for grafting. The competition of these two effects (insufficient charge and salting out) leads to an ideal charge ratio for maximising the grafting of SWCNTs.

However, by changing the SWCNT loading, the ideal charge ratio shifts due to the changing concentration of sodium. By plotting the degree of grafting as a function of sodium concentration (Fig. 5b), a consistent ideal sodium concentration of ∼25 mM can be identified. This effect has previously been seen for grafting of alkyl halides to exfoliated sodium graphite intercalation compounds,^50^ at a lower concentration of ∼10 mM. Despite the higher ionic concentration, the Debye length in the DMAc/SWCNT system is calculated to be 2.7 nm, greater than the value of 1.1 nm seen for exfoliated reduced graphite in THF, due to the higher dielectric constant of DMAc relative to THF (*ε*_THF_ = 7.5, *ε*_DMAc_ = 37.8); although the Debye lengths are similar (around 1–2 nm), the effect of changing geometry from planar to rod-like remains an open theoretical question.

### 3.3. Reductive purification of SWCNTs

It has been previously reported that (electro)chemical reductive dissolution of a SWCNT sample proceeds *via* initial dissolution of impurities and short defective nanotubes, followed by the SWCNTs at higher reduction potentials, providing a path to SWCNT purification.^24^ During reductive purification with NaNp/DMAc, the system potential was fixed by maintaining a fixed stoichiometry of sodium to SWCNT, analogous to the potentiostatic approach used in electrochemical purification.^55^ The NaNp/DMAc solution was simply poured over the dried SWCNTs; the solutions were not stirred in order maximise any kinetic difference in dissolution of impurities and SWCNTs. Increasing charge can be seen to lead to increasing dissolution of material, though a lack of stirring led to lower levels of dissolution than the stirred counterparts (Fig. 3).

**Fig. 6:**
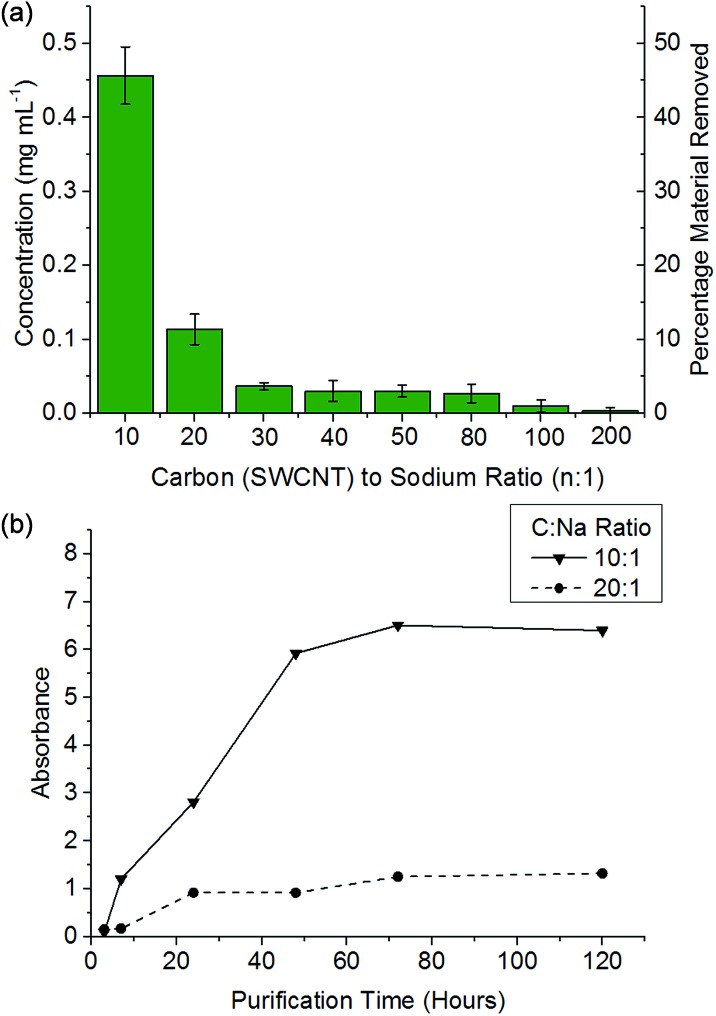
(a) Concentration of material dissolved *versus* C/Na after 48 h soaking of 1 mg mL^–1^ SWCNT in DMAc. (b) Absorbance (660 nm) of NaNp/DMAc and SWCNTs at a given SWCNT to sodium ratio *versus* time.

**Fig. 7:**
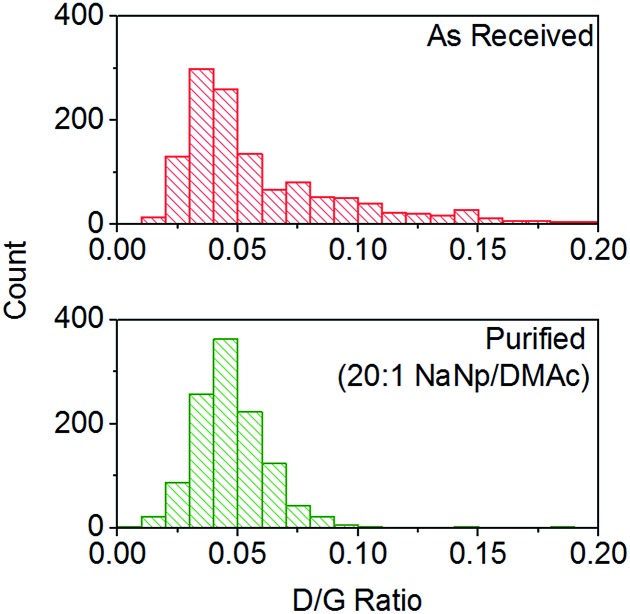
Distribution of D/G Raman ratios of Elicarb SWCNTs before and after NaNp purification (20 : 1 purification, 633 nm excitation). D/G of as received SWCNTs = 0.06 ± 0.037, D/G of as purified SWCNTs = 0.047 ± 0.015.

Purification with low levels of NaNp (≥30 : 1 C : Na) leads to the dissolution of small levels of material (Fig. 6) which TEM (ESI 5†) and SEM (ESI 6†) show is not CNTs; however, significant levels of carbonaceous impurities also remain in the purified fraction. Higher charge regimes increase the purity of the sample substantially, however at extreme charging regimes (≥10 : 1 C : Na) a significant proportion of the material is dissolved including the removal of pristine SWCNTs, illustrated by a reduction in the D/G Raman ratio of the dissolved fraction (ESI 7†). Furthermore, above 20 : 1 C/Na, the quality of the purified sample does not increase notably with similar levels of carbonaceous impurities seen for 20 : 1 and 10 : 1 fractions, despite a further 34 wt% of the initial SWCNT sample dissolving (Fig. 6a); to prevent unnecessary loss of material during purification, an optimum C : Na ratio (around 20 : 1 for Elicarb SWCNTs) should be applied. The purified materials (20 : 1) show a decrease in average the D/G ratio (Fig. 7), due to the removal of the highly defective materials; the distribution of D/G measurements around 0.05 remains similar, implying that remaining SWCNTs are undamaged. As higher concentrations and yields of nanotubide can be seen for stirred samples compared to fractions removed during the unstirred purification at similar stoichiometries, it can be assumed that the concentration values obtained from dissolution during purification are not due to saturation. The material dissolved in purification is thought to be driven kinetically with smaller impurities able to dissolve out of the bulk, driven in part by coulombic repulsion from the rest the sample. SWCNTs will be limited to reptation out of the network which is slow due to their high lengths (diffusion time of SWCNT reptation is inversely proportional to length cubed)^56^ leading to the majority of SWCNTs being kinetically trapped in the bulk network. Increasing the charge density of the system swells the network through coulombic repulsion, allowing longer SWCNTs to escape. In addition, while the charge density of a specific carbon/inorganic species will depend on its density of states and defect concentration, the distribution of sodium between dissolved and undissolved materials (ESI 10†) implies that the majority of charge remains in the undissolved material leading to the conclusion that the purification is not thermodynamically driven. By introducing stirring, the networks are physically agitated, and the concentration gradient between undissolved material surface and bulk solution reduced,^57^ accelerating the reptation and dissolution of the SWCNTs and lowering the difference in timescale between impurity and SWCNT dissolution. Here, the concentration of the material dissolved will be limited by saturation or percentage of material which is intrinsically soluble, as seen for standard dissolution of SWCNTs using DMAc/NaNp and stirring (Fig. 3). The purification of SWCNTs benefits subsequent functionalisation; the removal of impurities increases the degree of functionalisation (ESI 11†) by allowing a greater percentage of the charge added to the system to be held on the SWCNTs increasing the available charge for functionalisation, as well as removing carbonaceous debris known to hinder functionalisation.^58^

## 4. Conclusions

In summary, a quick, simple, one-step dissolution of SWCNTs has been demonstrated through the use of DMAc as a stable solvent for both sodium naphthalide and reduced SWCNTs, enabling the synthesis of concentrated solutions of nanotubide. Additionally, the inherent scalability of the NaNp/DMAc process provides a potentially large-scale source of individualised SWCNTs, of particular use for electronic devices and high surface area applications including electrochemical electrodes and catalyst supports. The concentrations of these solutions are notably high for individualised SWCNT solutions, particularly compared to the individualised fraction of ultracentrifuged SWCNT dispersions (<0.01 mg mL^–1^).^59^ However, the use of relatively large volumes of solvent may favour initial applications in high value/high performance applications of individualised SWCNTs, for example in thin films and devices. Higher concentrations may be obtained in future if a nematic phase can be isolated and/or with the addition of complexing agents for the sodium counter-ions.^60^

These solutions can be easily functionalised with a host of known reagents and the extent of grafting can be maximised by decreasing the steric bulk of the grafting species and using a leaving group with high polarisability. The extent of functionalisation can be seen to be a function of counter cation concentration with an idealized concentration of 25 mmol dm^–3^ giving a maximum grafting ratio. Additionally, modifying this method allows quick and simple access to reductive purification^55^ and the option to sequentially add further NaNp solution to directly dissolve the purified material. It is hoped that this work will facilitate and expand research in the versatile field of nanotubide chemistry by providing a relatively convenient route to their creation using cheap, available and easy to handle reagents.
