# Supplementary material for: A one-step route to solubilised, purified or functionalised single-walled carbon nanotubes
Source: J Mater Chem A Mater. 2015 Jul 23;3(32):16708–15. doi: 10.1039/c5ta03561a (PMC4786951; doi:10.1039/c5ta03561a)
Supplement: Supplementary file 1 [file TA-003-C5TA03561A-s001.pdf]

Electronic Supplementary Material (ESI) for Journal of Materials Chemistry A.
This journal is © The Royal Society of Chemistry 2015

Supporting Information

Debye lengths were calculated from $\kappa^{-1} = (\varepsilon_r \varepsilon_0 k_B T / 2\rho_\infty e^2)^{0.5}$ where ε is relative permeability (37.8 for DMAc, 7.58 for THF), ε_0 is the electric constant, k_B is Boltzmann constant, T is absolute temperature (room temperature of 278 K is assumed), p_∞ is number density of the ion in dm^{-3} and e is elemental charge.

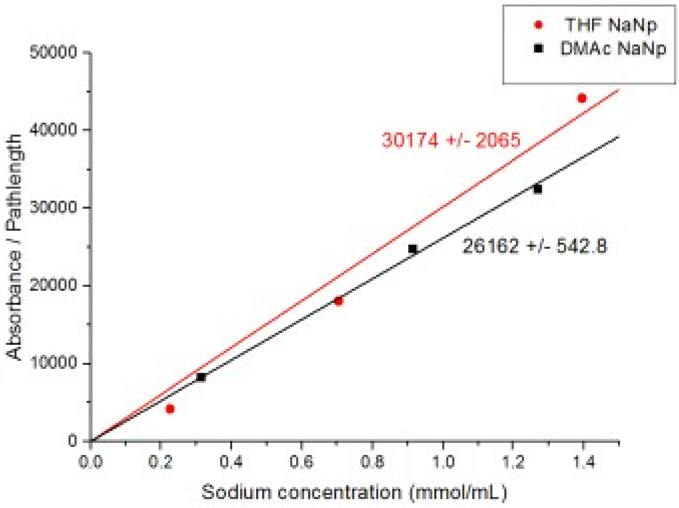

ESI 1. Extinction coefficient determination of NaNp in THF (761 nm) and DMAc (798 nm). Calculated using 100 times diluted samples (15 μL solution and 1485 μL solvent) and a 4 mm path length screw cap optical glass cuvette sealed with PTFE tape. Data fitted to pass through the origin and slopes are given next to the curves (standard errors supplied)

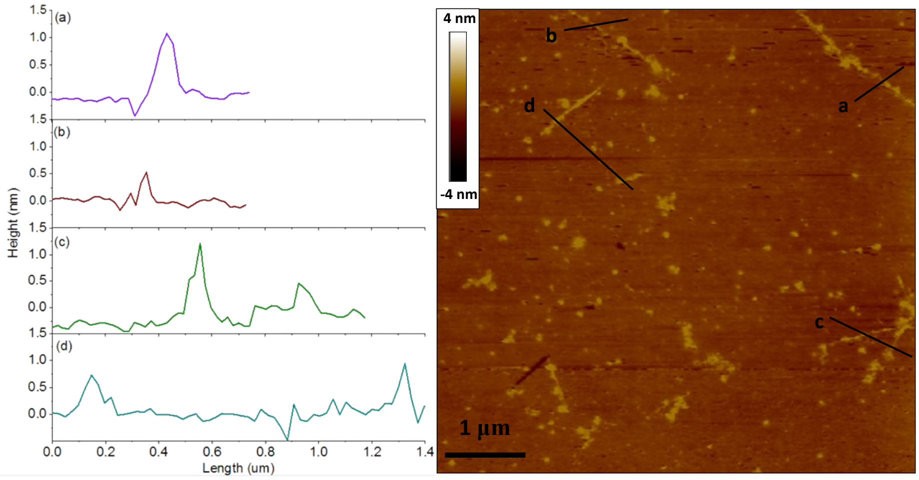

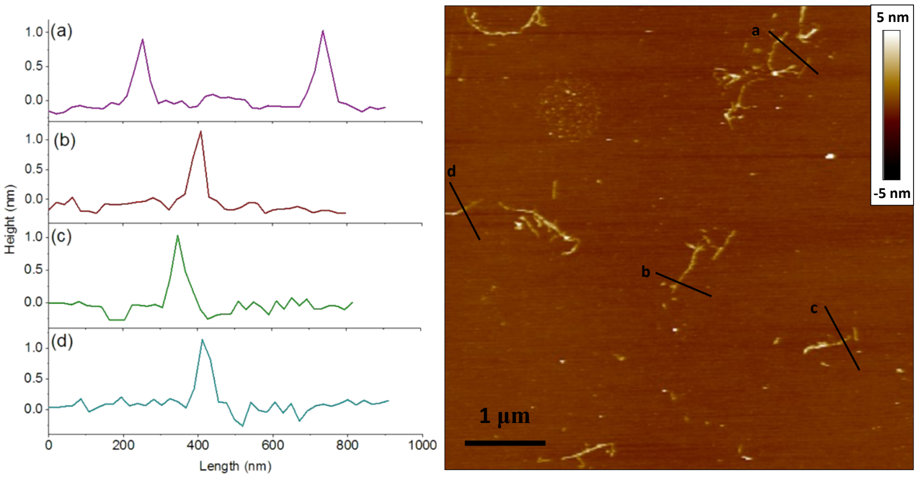

ESI 2. AFM of Elicarb SWCNTs dispersed with DMAc/NaNp (10:1); before (top) and after (bottom) reductive purification (20:1)

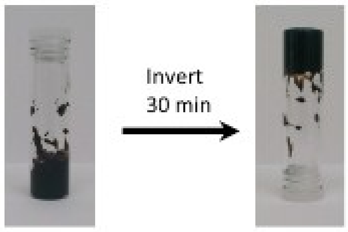

ESI 3. Picture of SWCNTs reduced at 10:1 C:Na in DMAc with a CNT system loading of 6.5 mg mL^{-1}

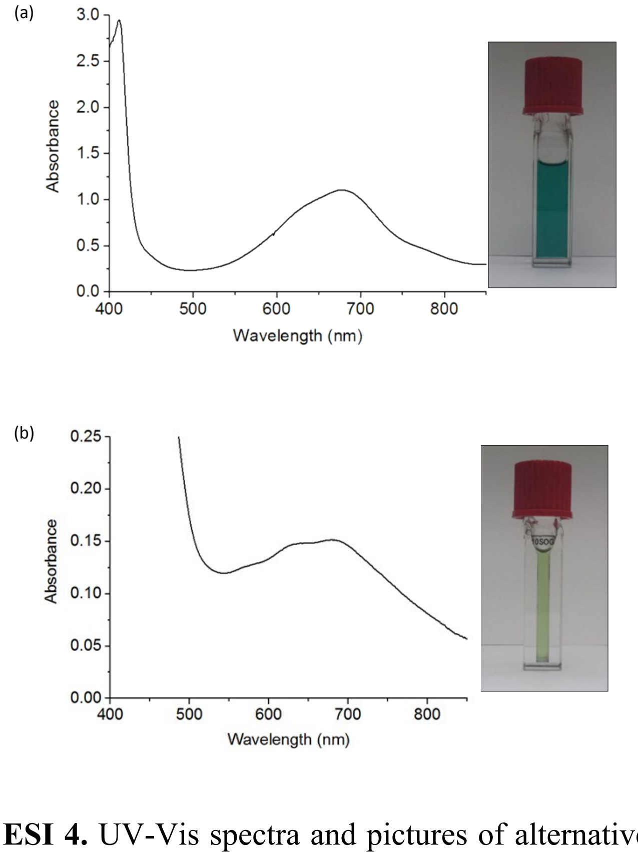

ESI 4. UV-Vis spectra and pictures of alternative single electron transport agents in DMAc. a) NaDTBP (0.2 mg(Na) mL^{-1}); b) KNp (0.1 mg(K) mL^{-1})

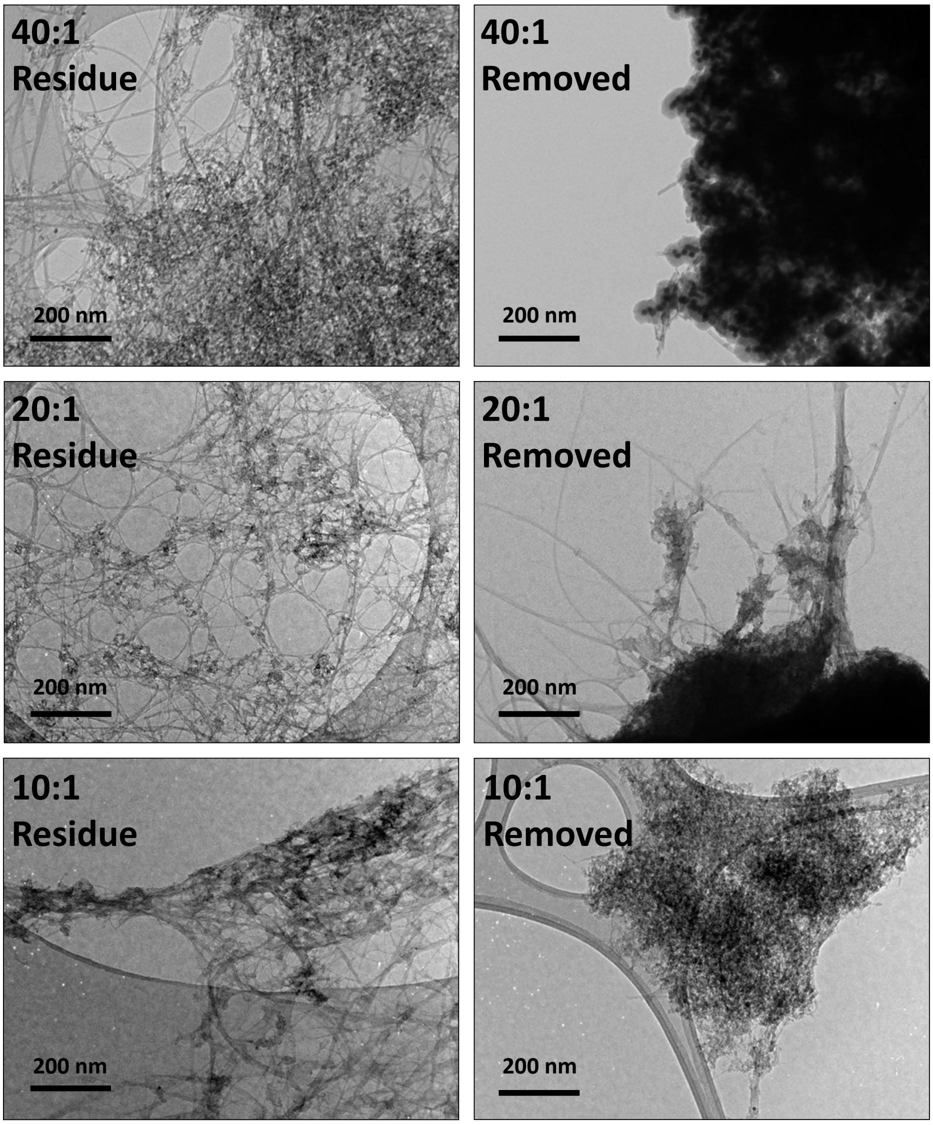

ESI 5. TEM of Elicarb purified nanotubes (left) and material removed (right)

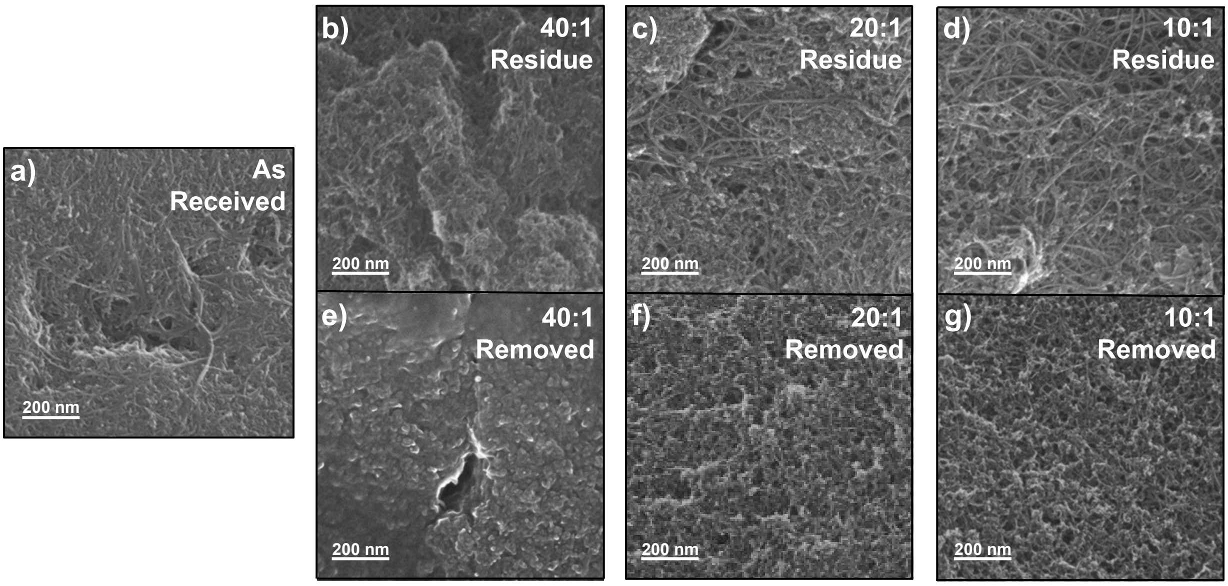

ESI 6. SEM of (a) as received SWCNTs, (b-d) Post-purified SWCNTs using b) 40:1 c) 20:1 and d) 10:1, and (e-g) Materials removed from purifications using e) 40:1 f) 20:1 and g) 10:1

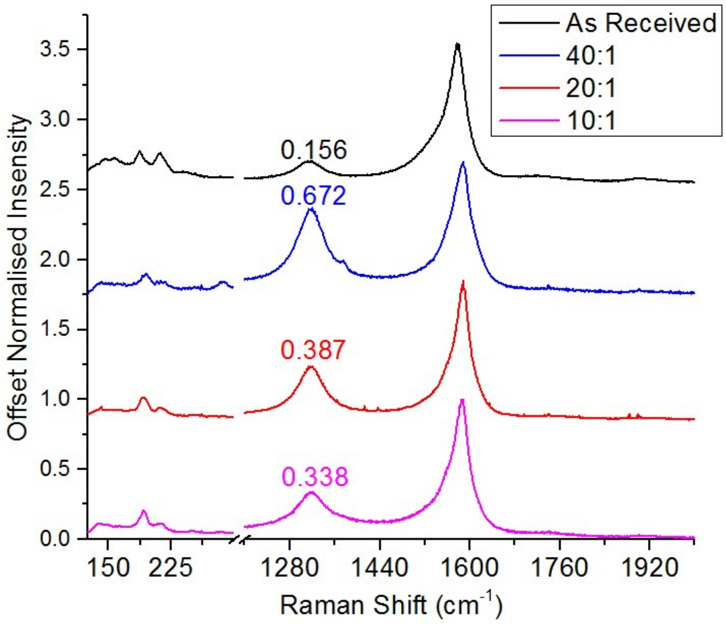

ESI 7. Raman (633 nm excitation) of as-received SWCNTs and filtered supernatant from different C:Na stoichiometry purifications after 48 h.

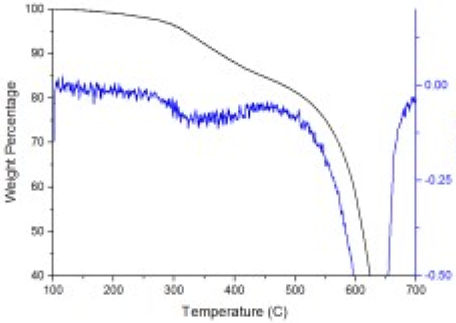

ESI 8. TGA and derivative of Elicarb-C_4H_9 demonstrating the separation of alkyl and CNT degradation at 460 ^{0}C. Mass of alkyl fragments are measured from weight loss between 100 and 460^{0}C.

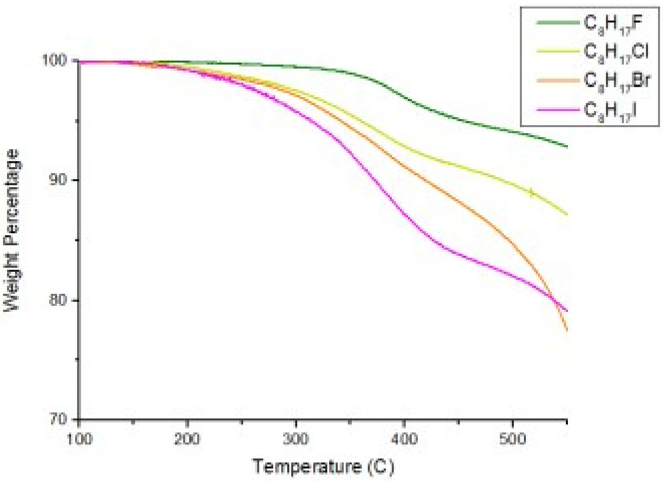

ESI 9. Typical TGAs of alkyl halide functionalised SWCNTs across an array of halides.

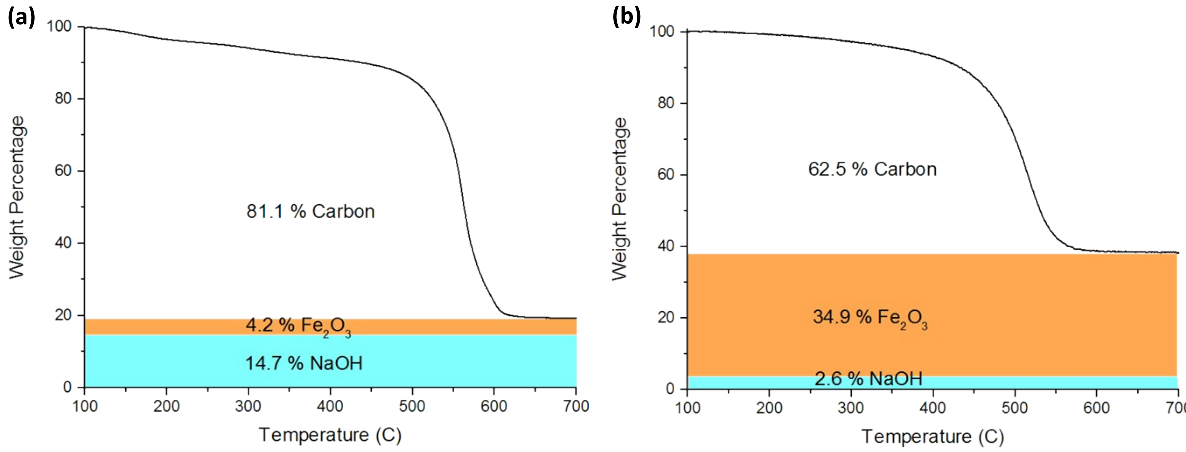

	EDX (atom %)		TGA (wt. %)		NaOH (wt. %)	Fe_2O_3 (wt. %)	Molar Ratio (normalised to Na)		
	Na	Fe	Carbon	Inorganic			Na	C	Fe
As-Received SWCNTs †			92.5	7.5	0	7.5	n/a	1	0.01
SWCNTs and Sodium ‡			79.3	20.7	6.4	14.3	1	18.5	0.23
Purified SWCNTs	63.6	36.4	81.1	18.9	12.0	6.9	1	22.5	0.29
Removed Impurities	13.1	86.9	62.5	37.5	4.9	32.6	1	42.4	3.32

ESI 10. Oxidative TGA of a) unwashed purified Elicarb SWCNTs and b) material removed during purification (20:1), and table of relative Fe/Na atomic percentages from EDX (and calculated weight percentages and ratios). EDX was measured on post-TGA residue with atomic ratios calculated only between iron and sodium to eliminate contamination of scattered signal. Using the ratio of material dissolved during the purification (11 wt. %, Fig 6a, main text), the sum total iron content measured from TGA/EDX of dissolved and residual materials from purification can be calculated as 10.7 wt. % of the initial SWCNTs, close to the known value of 7.5 wt. % for as received SWCNTs demonstrating the validity of this test.

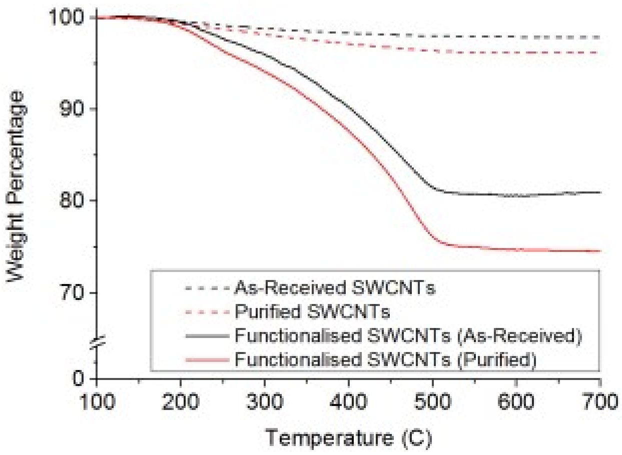

ESI 11. TGA of as-received and purified Elicarb before and after, reductive functionalisation with 1-iodododecane. Nanotubes purified using 20:1

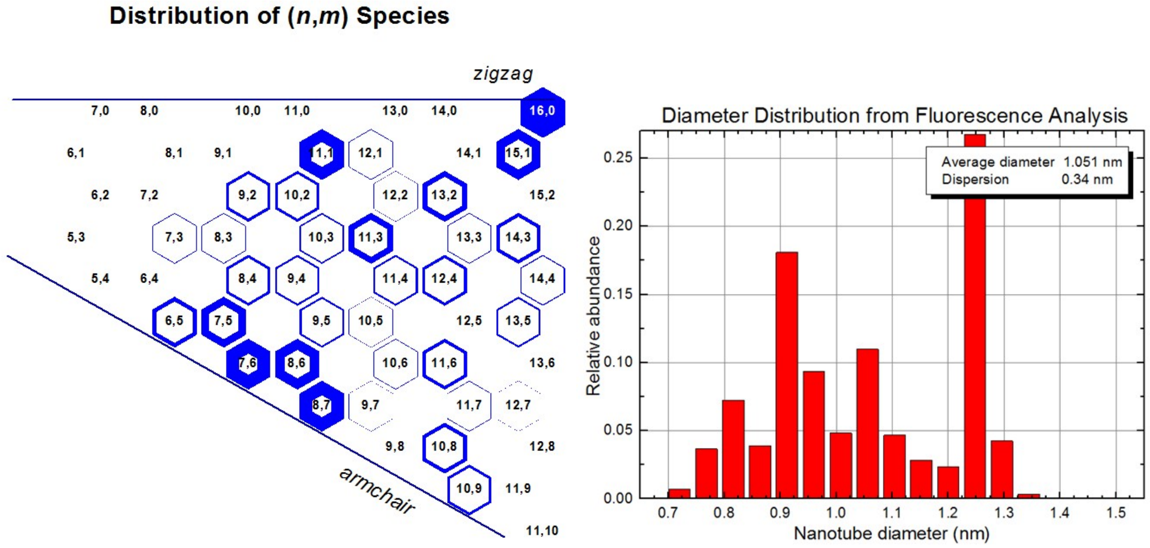

ESI 12. Chirality and diameter distributions of Elicarb SWCNTs (PR929, batch 108511/g). Nanotubes were probe sonicated in DOC (1 wt%) in D_2O (~1 mg mL^{-1}) and centrifuged at 42,000 *g* for 45 min. Measurements were taken with a Model NS1 NanoSpectralyzer (Applied NanoFluorescene, LLC) with data fitted to a preinstalled HiPco model.
